# Chromosome-level genome assembly of watershield (*Brasenia schreberi*)

**DOI:** 10.1038/s41597-023-02380-z

**Published:** 2023-07-19

**Authors:** Bei Lu, Tao Shi, Jinming Chen

**Affiliations:** 1 Aquatic Plant Research Center, Wuhan Botanical Garden, Chinese Academy of Sciences, Wuhan, 4300074 China; 2 University of Chinese Academy of Sciences, Beijing, 100049 China

**Keywords:** Plant evolution, Conservation biology

## Abstract

Watershield (*Brasenia schreberi*) is an aquatic plant that belongs to the basal angiosperm family Cabombaceae. This species has been cultivated as an aquatic vegetable for more than 3000 years in East Asia, but the natural populations have greatly declined in recent decades and have become endangered in several countries of East Asia. In this study, by using PacBio long reads, Illumina short reads, and Hi-C sequencing data, we assembled the genome of *B*. *schreberi*, which was approximately 1170.4 Mb in size with a contig N50 of 7.1 Mb. Of the total assembled sequences, 93.6% were anchored to 36 pseudochromosomes with a scaffold N50 of 28.9 Mb. A total of 74,699 protein-coding genes were predicted in the *B. schreberi* genome, and 558 Mb of repetitive elements occupying 47.69% of the genome were identified. BUSCO analysis yielded a completeness score of 95.8%. The assembled high-quality genome of *B. schreberi* will be a valuable reference for the study of conservation, evolution and molecular breeding in this species.

## Background & Summary

**Fig. 1:**
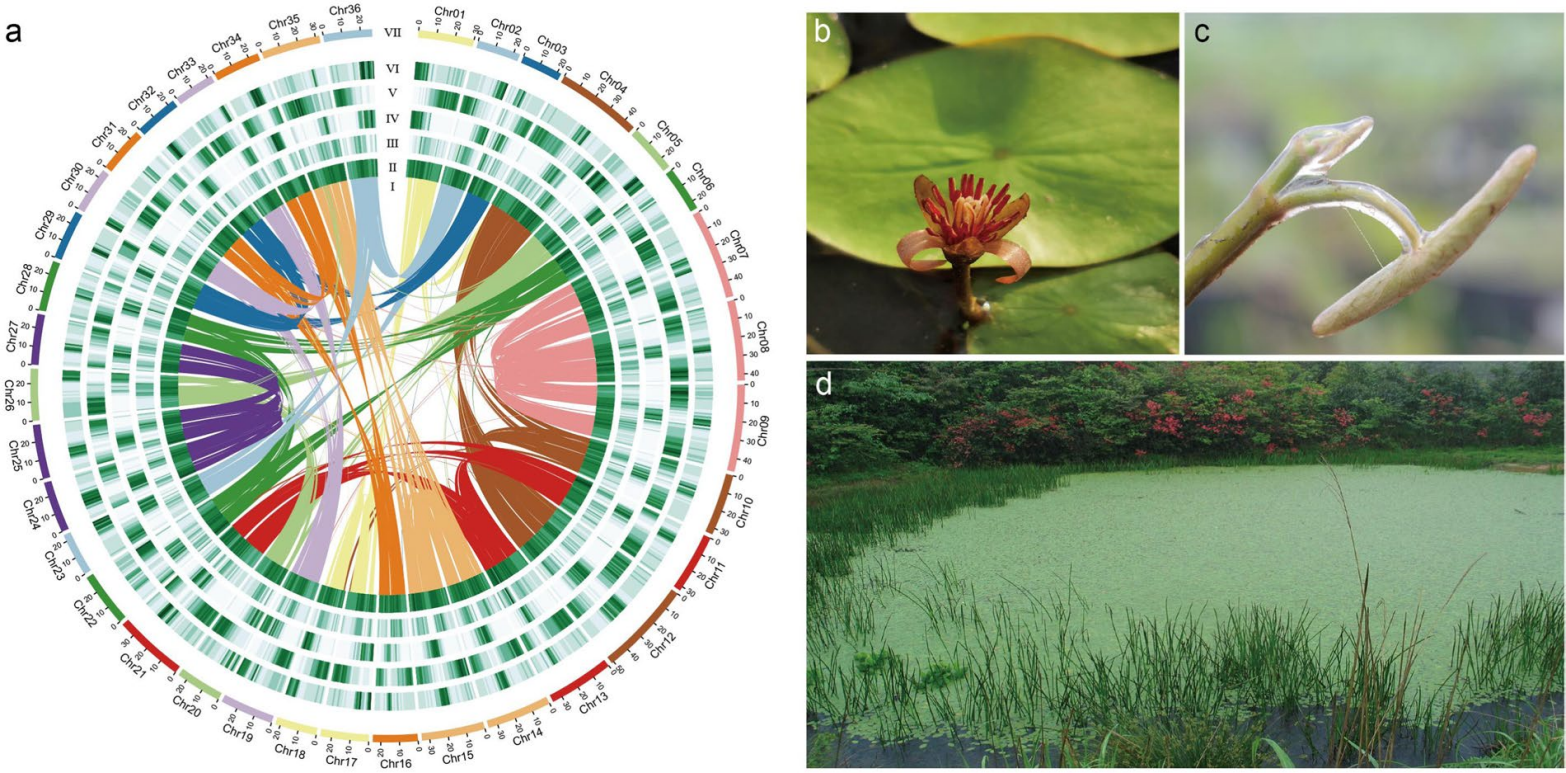
Overview of the genome and plants of *Brasenia schreberi*. (**a**) Circos plot of anchored *B. schreberi* genomic features. Difference tracks show: (I) Links of intragenomic syntenic blocks, only blocks with more than five syntenic genes are shown, (II) LTR/Copia density in 1 Mb sliding windows, (III) LTR/Gypsy density in 1 Mb sliding windows, (IV) repeat density, (V) gene density in 1 Mb sliding windows (minimum-maximum, 0–30), (VI) GC content in 1 Mb sliding windows. (**b**) A flower on its second day of blossoming. (**c**) Juvenile leaf and bud with mucilage covering. (**d**) A natural population during the growing season.

*Brasenia schreberi* J.F. Gmel. (watershield) is a monotypic species in the genus *Brasenia* (Cabombaeae), which belongs to the basal angiosperm order Nymphaeales. This species is a perennial floating leaved freshwater aquatic plant that is found in the tropical and temperate regions of America, Africa, Australasia, and Asia^1^. *B. schreberi* produces thick mucilage that covers the juvenile leaf abaxial surface and buds^2,3^ (Fig. 1). This mucilage has been found to have anti-algal and antibacterial properties and may function as an herbivory defense, protecting young buds from abrasion, and as an excellent biological lubricant^4^. *B. schreberi* has been cultivated as an aquatic vegetable for more than 3000 years in East Asia due to the importance of mucilage-covered young leaves and buds in the diet and the special flavor of the mucilage^5^.

Plant mucilage is a gelatinous matrix comprising mostly polysaccharides known as pectins produced by glandular trichomes (GTs), seed coats, root hairs, etc.^6,7^, serving various functions for plants. Although all investigated lineages of the basal angiosperm order Nymphaeales possess epidermal trichome-like structures (GTs), only a few species, such as *B. schreberi*, have a mucilage layer secreted by the GTs^7–9^. Thus, *B. schreberi* represents an interesting system for studying the evolution and molecular mechanisms of plant mucilages. In addition, plant GTs have been important target traits for crop breeding^10^.

In the past three decades, due to human activity and habitat loss, the natural populations of *B. schreberi* have decreased significantly and are considered endangered in several counties of East Asia^11,12^. For example, in China, this species has been previously listed as the first category of key protected wild plants^13^; in Korea, it is listed as a critically endangered species^14^. For conservation purposes, several population genetic studies using few molecular markers have been conducted on *B. schreberi* in China and Korea^11,12,15,16^. However, these studies utilized only limited regions of the genome, and further conservation genomics studies at the whole genome scale are needed to establish effective management strategies for this endangered species in Asia.

**Fig. 2:**
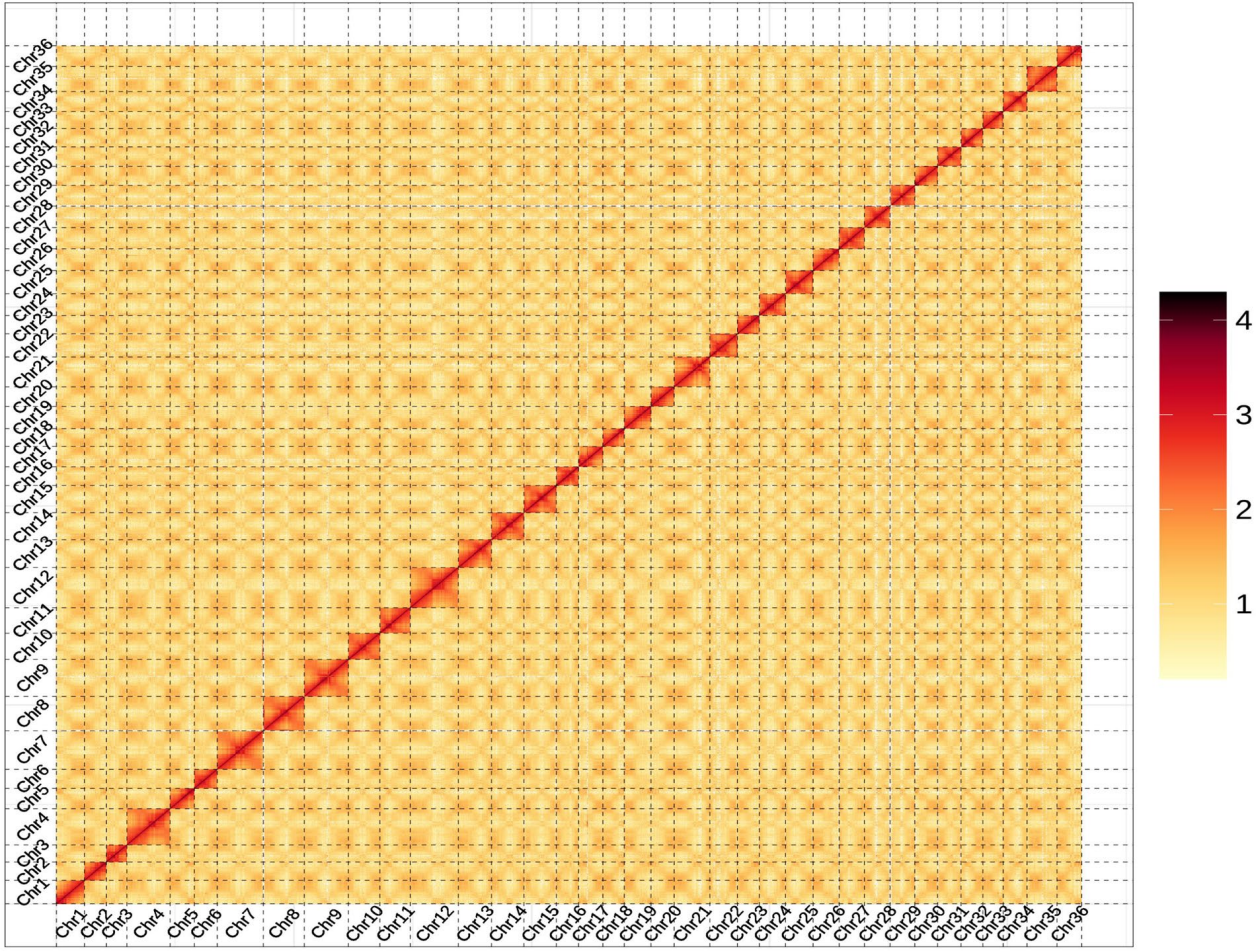
Hi-C interaction heatmap within pseudochromosomes of *Brasenia schreberi*.

In this study, we presented a high-quality genome sequence for *B. schreberi* obtained using PacBio, Illumina, and Hi-C technologies (Fig. 1). The assembled genome had a size of 1,170.4 Mb with a contig N50 of 7.1 Mb and a scaffold N50 of 28.9 Mb (Table 1). The assembled scaffolds were further anchored to 36 pseudochromosomes, with an anchoring rate of 93.6% (Fig. 2, Table 1). A total of 74,699 protein-coding gene models were fully annotated (Table 2). Repetitive elements (TEs), with a collective length of 558 Mb, occupied 47.69% of the *B. schreberi* genome (Table 2). The quality of the final genomic assembly was assessed to be high gene completeness (95.8%), as indicated by BUSCO^17^. The assembled high-quality genome of *B. schreberi* should be a valuable resource for future conservation genomics studies. In addition, our assembled reference genome of this basal angiosperm offers a new resource for studying the origin and early adaptive evolution of angiosperms and for revealing the molecular basis of the trait of mucilage secretion, which will facilitate molecular breeding in this aquatic vegetable.

**Table 1:** Summary of *Brasenia scherberi* genome assembly.

Genome assembly statistics	
Total length of Contigs (Mb)	1,170.4
Number of Contigs	3,846
N50 length of contigs (Mb)	7.1
Longest contig (Mb)	51.2
Number of scaffolds	2,173
N50 length of scaffolds (Mb)	28.9
Anchored rate (%)	93.6
BUSCO score (Eukaryota) (%)	95.8
LTR Assembly Index, LAI	6.21

**Table 2:** Summary of *Brasenia scherberi* genome annotations.

Genome assembly statistics	Count	Proportion in genome (%)
Predicted protein-coding genes	74,699	
NR	61,366	82.15
pfam	47,317	63.34
KEGG	29,431	39.40
GO	41,427	55.46
eggNOG	55,144	73.82
Annotated	61,618	82.49
Percentage of repeat elements	1,123,230	47.69
Copia	165,769	11.23
Gypsy	156,404	14.18

## Methods

### Sampling, sequencing and genome size estimation

*B. schreberi* plants were originally collected from a natural population in Lichuan, Hubei Province, China, in 2018 and cultivated in the Wuhan Botanical Garden (WBG) of the Chinese Academy of Sciences. After the collection of juvenile leaves, the mucilage on the back of the leaves was washed off, and the leave samples were promptly stored in liquid nitrogen. Then the high-quality genomic DNA was extracted from the processed samples using the MagicMag plant genomic DNA Micro Kit (Sangon Biotech Co.) and used for subsequent Illumina and PacBio sequencing. The MGI libraries were constructed and sequenced on a DNBSEQ-T7 platform at an expected coverage of 80 × (see table deposited at Figshare^18^). The MGI short reads were used for both genome size estimation and residual error correction in the *de novo* genome assembly. For PacBio sequencing, 20 kb DNA libraries were constructed and then sequenced using single molecule real-time (SMRT). A total of 101 Gb of data composed of 5.4 million subreads were generated on the PacBio Sequel platform (Pacific Biosciences) (see table deposited at Figshare^18^). The Hi-C libraries were constructed following a previously published protocol^19^. The sample underwent liquid nitrogen grinding and was then cross-linked with 4% formaldehyde at room temperature under vacuum for 30 minutes. Quenching of the crosslinking reaction was achieved by adding 2.5 M glycine for 5 minutes followed by incubation on ice for 15 minutes. After centrifugation at 2500 rpm and 4 °C for 10 minutes, the pellet was washed with 500 μl PBS, centrifuged again, and resuspended in a lysis buffer. The resulting supernatant was subjected to further centrifugation, and the pellet was washed, resuspended, and solubilized using dilute SDS at 65 °C for 10 minutes. Subsequent steps involved digestion with a 4-cutter restriction enzyme DpnII overnight at 37 °C, marking of DNA ends with biotin-14-dCTP, and blunt-end ligation of cross-linked fragments. Proximal chromatin DNA was re-ligated, nuclear complexes were reversely cross-linked with proteinase K at 65 °C, and DNA was purified by phenol-chloroform extraction. Biotin was removed from nonligated fragment ends, and sheared fragments were repaired. Biotin-labeled Hi-C samples were enriched using streptavidin C1 magnetic beads. After addition of A-tails and ligation with Illumina PE sequencing adapters, Hi-C libraries were PCR-amplified (12–14 cycles) and sequenced on Illumina PE150 platform at Novogene Biotech Co., Ltd. (Beijing, China) for chromosome construction.

For genome functional annotation, transcriptome sequencing was performed with seven tissues of *B. schreberi*, including stamen, pistil, perianth, stem, root, rhizome, and leaf. RNA libraries were prepared using the TruSeq RNA Sample Prep Kit (Illumina, USA) according to the manufacturer’s instructions, and PE150 sequencing was conducted on an Illumina NovaSeq. 6000 platform at Novogene Biotech Co., Ltd. (Beijing, China).

The genome size and ploidy levels of *B. schreberi* were estimated using two methods: (i) flow cytometry, which was conducted on a BD AccuriTMC6 flow cytometer (BD Biosciences) using the leaf of *Nelumbo nucifera* (genome size ≈ 807.6 Mb^20^) as a reference, and the genome size of *B. schreberi* was estimated as ~1100 megabases (Mb) by this method (see figure deposited at Figshare^18^); and (ii) *k*-mer-based estimation, in which the *k*-mer distribution of Illumina reads was counted by using jellyfish v2.3.0 (*k*-mer = 21, parameters: count -m 21 -t 10 -s 1 G), and then the genome size and the rate of heterozygosity were estimated to be ~956.2 Mb and 0.10%, respectively, by GenomeScope online version (http://qb.cshl.edu/genomescope/) using the *k*-mer count distribution file (see figure deposited at Figshare^18^); ploidy levels were assessed by Smudgeplot v0.2.5^21^ based on heterozygous *k*-mer pairs (see figure deposited at Figshare^18^).

### Genome assembly

Canu v.1.8^22^ (parameters: -p out genomeSize = 1.5 g maxThreads = 30 useGrid = false) was used for self-correction, trimming, and assembly. To polish the draft assembly, PacBio subreads were subjected to three rounds of polishing with the program Racon v1.4.3 (https://github.com/isovic/racon), and then the Illumina paired-end reads were further subjected to three rounds of polishing with the program Pilon v1.23^23^ (parameters: --fix all --changes). Finally, the total length of the draft genome was 1170.4 Mb, comprising 3,846 contigs with a contig N50 of 7.1 Mb and a maximum length of 51.2 Mb (Table 1).

The assembly was refined using high-throughput chromosome conformation capture (Hi-C) data. The 2,802,461 Hi-C paired-end reads, which were grouped to the chromosome level using ALLHiC^24^, were remapped to the draft assembly. We divided the assembled chromosomes into equally sized bins (500 Kb) and constructed an interaction heatmap based on the number of valid paired-end reads supporting interactions between each pair of bins. Then, the visual correction of assembly was finalized using JuiceBox v.2.1.10^25^ based on the intensity of chromosome interaction (Fig. 2). The specific criteria we employed for visual correction were as follows: We adjusted the assembly based on the principle that intra-chromosomal interactions should be stronger than inter-chromosomal interactions. If there were evident assembly errors within a completed contig, we would break and adjust it according to the interaction relationships. Additionally, very short contigs without any interaction relationships were placed in the unassigned category. The chromosome-level genome assembly was improved, containing 2,173 scaffolds with a scaffold N50 of 28.9 Mb (Table 1). These scaffolds were further anchored onto 36 pseudochromosomes^26–28^, resulting in a total of 36 chromosomes and 2137 additional scaffolds, with an anchor rate of 93.6% (Table 1).

### Genome annotation

First, the EDTA (Extensive *de novo* TE Annotator) program v2.0.1^29^ was used to annotate the repeat sequences, including the repetitive element (TE) sequences, and generate the masked repeat sequence for gene prediction. Repetitive elements with a collective length of 558 Mb occupied 47.69% of the *B. schreberi* genome (Table 2). Then, three algorithms were used to predict genes: *ab initio*, homolog, and transcriptome alignment. Seven RNAseqs generated in this study were used in both methods of transcriptome-based alignment: (1) Transcriptomes were assembled by Trinity v2.5.1^30^ (both *de novo* and guided), and the PASA (Program to Assemble Spliced Alignments v2.3.3)^31^ program was used to align these redundant transcriptomes to genome sequences; (2) transcriptomes were assembled from a hisat2-stringtie pipeline^32^, and the open reading frames (ORFs) were predicted by TransDecoder v5.5.0 (https://github.com/TransDecoder/TransDecoder/). Homolog protein comparison was conducted using GETA v2.4.12 (https://github.com/chenlianfu/geta) (parameters: homolog_genewise–cpu 40–coverage_ratio 0.4–evalue 1e-9–max_gene_length 2000) with the program GeneWise^33^. The *ab initio* method was Braker2^34^. The EVidenceModeler program (EVM-1.1.1)^35^ was used to integrate the above redundant annotation information. Three rounds of PASA annotation updates were performed to obtain annotation information for the genome. Combining the *ab initio*, RNA-seq, and homology-based methods, a total of 74699 protein-coding gene models were fully annotated (Table 2). The predicted gene length overlap larger than 30% with repeat sequences was filtered by TransposonPSI v1.0.0 (http://transposonpsi.sourceforge.net/) for the downstream analysis. For functional annotation, we performed searches of our predicted protein-coding genes against the non-redundant (NR) using BLASTP v2.9.033^36^, Pfam, Gene Ontology (GO), Kyoto Encyclopedia of Genes and Genomes (KEGG) databases, as well as the eggNOG database using EggNOG-mapper^37^ v2.1.11 (Table 2). The completeness 82.15% of protein-coding genes had significant hits in the functional annotation databases (Table 2).

### Orthologue and phylogenetic analyses

**Fig. 3:**
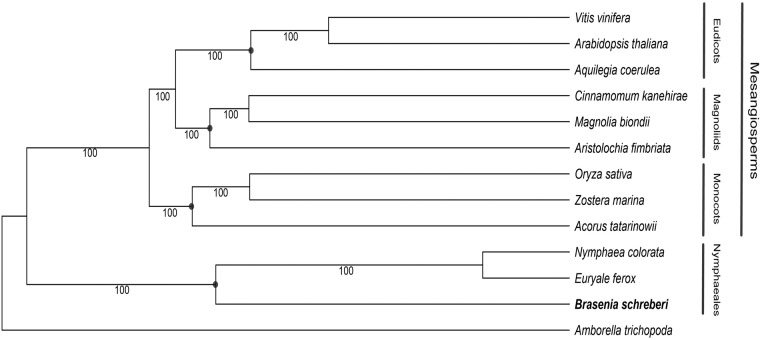
Phylogenetic tree of 13 angiosperm species generated by RAxML, number below branches displaying bootstraps support of 100%.

The genome protein sequences of 13 angiosperms were used to determine the orthologs using OrthoFinder v2.5.4^38^, including three Nymphaeales (*B. schreberi*, *Nymphaea colorata*, and *Euryale ferox*), three monocots (*Acorus tatarinowii*, *Zostera marina*, and *Oryza sativa*), three magnoliids (*Aristolochia fimbriata*, *Cinnamomum kanehirae*, and *Magnolia biondii*), three eudicots (*Aquilegia coerulea*, *Vitis vinifera*, and *Arabidopsis thaliana*), and *Amborella trichopoda* (see table deposited at Figshare^18^). The resulted 158 single-copy orthologs were aligned using MAFFT v7.505 with default settings^39^. The corresponding nucleotide sequence alignments of the protein alignments were extracted using pal2nal.pl v14^40^ and trimmed with Gblocks v0.91b^41^ with the codon model. The maximum likelihood tree was constructed under ‘GTRGAMMA’ model of nucleotide substitution using RAxML v8.2.12^42^ with 100 bootstrap replicates (Fig. 3).

## Data Records

The raw data of MGI and Hi-C sequencing were submitted to the National Center for Biotechnology Information (NCBI) Sequence Read Archive database with accession numbers SRR24223717^43^, and SRR24223715^44^. Seven transcriptome data were submitted to NCBI with accession numbers SRR24136212^45^, SRR24136211^46^, SRR24136210^47^, SRR24136209^48^, SRR24136208^49^, SRR24136207^50^, SRR24136206^51^, under the BioProject accession number PRJNA954463. The genome assembly data, genome annotation files, gene CDS, and protein data have been deposited into CNGB Sequence Archive (CNSA)^52^ with the accession number CNA0069000 under the BioProject accession number CNP0004217. The raw PacBio sequences have been deposited into CNSA^52^ under the BioProject accession number CNP0004217 (https://ftp.cngb.org/pub/CNSA/data5/CNP0004217/CNS0724876/CNX0616196/CNR0710381/). The genome annotation files had also been deposited at the Figshare^53^. The genome genome assembly data had also been submitted to GenBank with accession number JARYZE000000000^54^.

## Technical Validation

Three methods were used to evaluate the quality of the genome assembly. First, in the Benchmarking Universal Single-Copy Orthologs (BUSCO, v5.2.2)^17^ evaluation, complete and single-copy, complete and duplicated, fragmented, and missing categories accounted for 44.1% (712), 51.7% (835), 1.9% (30), and 2.3% (37) of 1614 Eukaryota BUSCO genes identified in the chromosome-level genome assembly, respectively. Then, we calculated the long terminal repeat (LTR) assembly index (LAI) based on the EDTA results with the default settings. The LTR Assembly Index (LAI) is used as a validation measure to assess the quality of LTR sequences in genome assembly. LAI calculates the number of correctly positioned LTRs and considers the integrity of LTR sequences. Higher LAI values indicate better positioning and integrity, implying higher assembly quality and accuracy of LTR sequences. It complements other assembly quality metrics, providing a comprehensive evaluation of the assembly outcomes. We also calculated the mapping rate of seven transcriptomes generated from different tissues and developmental stages (see table deposited at Figshare^18^). In total, the quality of the final genomic assembly was assessed to be high completeness (95.8% indicated by BUSCO), contiguity (6.21 indicated by LAI), and consistency (97%~98% mapping rate of RNA-seq datasets).

## Data Availability

No custom programming or coding was used.
